# Trends in the Cost and Utilization of Publicly Reimbursed Cancer Medications Dispensed as Take-Home Treatments from 2017–2021

**DOI:** 10.3390/curroncol32040237

**Published:** 2025-04-18

**Authors:** Ria Garg, Tara Dumont, Daniel McCormack, Mina Tadrous, Tonya Campbell, Kelvin Chan, Tara Gomes

**Affiliations:** 1 Leslie Dan Faculty of Pharmacy, University of Toronto, Toronto, ON M5S 3M2, Canada; ria.garg@mail.utoronto.ca (R.G.); tara.dumont@mail.utoronto.ca (T.D.); mina.tadrous@utoronto.ca (M.T.); 2 ICES, Toronto, ON M4N 3M5, Canada; daniel.mccormack@ices.on.ca (D.M.); kelvin.chan@sunnybrook.ca (K.C.); 3 Li Ka Shing Knowledge Institute, St. Michael’s Hospital, Toronto, ON M5B 1T8, Canada; tonya.campbell@unityhealth.to; 4 Institute for Health Policy, Management and Evaluation, University of Toronto, Toronto, ON M5T 3M6, Canada; 5 Sunnybrook Health Sciences Centre, University of Toronto, Toronto, ON M4N 3M5, Canada

**Keywords:** outpatient cancer treatment, take-home cancer medications

## Abstract

**Background:** The cost and uptake of cancer medications dispensed as take-home treatments are not well understood. Therefore, in this study, we describe trends and the impact of SARS-CoV-2 on the utilization and cost of take-home cancer medications dispensed through the public payer system in Ontario, Canada. **Methods:** We conducted a repeated cross-sectional time-series analysis examining monthly and fiscal-year trends in the utilization and cost of take-home cancer medications reimbursed by the public payer between 1 April 2017 and 31 March 2021, in Ontario, Canada. Our primary outcome was per-beneficiary spending. Total public payer spending and the number of unique beneficiaries who were dispensed take-home cancer medications were reported as secondary outcomes. All outcomes were reported overall and stratified by drug class. We used autoregressive integrated moving average (ARIMA) models to assess the impact of the SARS-CoV-2 pandemic on the aforementioned trends. **Results:** Annual per-beneficiary spending on take-home cancer medications increased by 32.8% (from CAD 4422 in 2017/18 to CAD 6579 in 2020/21) over the study period. The rise in per-beneficiary spending was driven by the cost of medications within the small-molecule targeted therapy and immunotherapy drug classes, which accounted for three-quarters of total public payer spending on take-home cancer medications in 2020/21 despite being dispensed to less than 8% of beneficiaries. Upon the declaration of emergency for SARS-CoV-2, a short-term decline in per-beneficiary spending (CAD −179 per month; *p*-value < 0.01) was observed between March and June 2020. This temporary decline was driven by an increase in the number of beneficiaries (5582 per month; *p*-value < 0.01) receiving low-cost take-home cancer medications within the cytotoxic chemotherapy and hormonal therapy drug class without a corresponding rise in public payer spending. **Conclusion:** Future research should investigate barriers to the widespread uptake of take-home cancer medications during periods of public emergencies, particularly for high-cost drugs.

## 1. Introduction

Cancer is a leading cause of death, accounting for 25% of all fatalities in Canada [1]. Over the past decade, numerous medications have entered the market that have improved cancer prognosis and patients’ quality of life while on treatment [2,3]. In addition to their therapeutic benefits, these medications may be administered either in-person or as take-home treatments. The simplified administration of take-home medications is associated with improved quality of life and reduced use of hospital infrastructure [4,5]. Despite these benefits, public payer reimbursement of take-home cancer medications varies significantly across Canadian provinces [6]. In Ontario, the public payer reimburses take-home cancer medications for patients insured under the provincial Ontario Drug Benefit (ODB) program, which includes individuals aged 65+, those who receive income or disability assistance, and those less than 25 years old without private insurance [7]. In contrast, individuals ineligible for ODB must navigate a mix of different reimbursement options (e.g., private insurance, patient support programs and out-of-pocket payments) before they may receive public payer coverage [7].

In March 2020, the declaration of emergency for SARS-CoV-2 led to the rapid transformation of cancer care delivery due to implementation of public health restrictions, which included reduced capacity for the provision of inpatient healthcare services and cancellation of non-urgent surgeries [8]. During the first wave of the SARS-CoV-2 pandemic, Ontario reported a 27.8% reduction in surgical cancer resections with preference being given to neoadjuvant therapy [9]. In situations where in-person administration of medication is not feasible, take-home cancer treatments may serve as a suitable alternative. Over the past decade, the ODB program’s spending on take-home medications indicated for cancer treatment has nearly tripled in Ontario, increasing from CAD 264 million in 2012 to CAD 988 million in 2022 [10]. However, it remains unknown whether the declaration of emergency for SARS-CoV-2 and the changing dynamics of cancer care delivery have further accelerated this trend and increased the number of OBD beneficiaries dispensed take-home cancer medications. Therefore, the objective of our study was to characterize trends and assess the impact of a declaration of emergency for SARS-CoV-2 on the uptake and cost of take-home cancer medications reimbursed by public payers in Ontario, Canada.

## 2. Methods

### 2.1. Study Design

We conducted a repeated cross-sectional interrupted time-series analysis examining monthly and fiscal year trends in the uptake and cost of take-home cancer medications reimbursed by the ODB Program between 1 April 2017 and 31 March 2021, in Ontario, Canada.

### 2.2. Data Sources

We used Ontario’s routinely collected health administrative databases, which are securely linked using unique, encoded identifiers and analyzed at ICES (formerly known as the Institute for Clinical Evaluative Sciences), an independent, nonprofit research institute whose legal status under Ontario’s health information privacy law allows it to collect and analyze health care and demographic data, without consent, for health system evaluation and improvement. Databases used in this study include the ODB database, which captures information (e.g., total cost reimbursed by the public payer) for all prescriptions dispensed through the ODB program at a community pharmacy. Eligibility for ODB coverage includes social assistance, high prescription drug costs relative to net household income, receipt of home care services, residence in a long-term care facility, being <25 years old without private insurance, and being ≥65 years old [11]. The ODB program provides reimbursement for all medications listed on the provincial formulary and those approved for reimbursement through the Exception Access Program [12]. The Registered Person’s Database (RPDB) was used to capture demographic data, location of residence, and vital statistics for all Ontario residents eligible for the universal Ontario Health Insurance Plan (OHIP). The OHIP Claims database was used to identify outpatient physician visits and patient comorbidities. The Canadian Institute for Health Information (CIHI) National Ambulatory Care Reporting System (NACRS), Same Day Surgery (SDS), and Discharge Abstract Databases (DAD) were used to capture diagnoses and procedures recorded during emergency department visits, outpatient surgeries, and inpatient hospitalizations, respectively. The Ontario Cancer Registry (OCR), the Activity Level Reporting (ALR) database, and the New Drug Funding Program (NDFP) database were used to identify outpatient oncology clinic visits, appointments for radiation or systemic therapy, and systemic chemotherapy medications administered in a hospital, respectively. Lastly, we used the ICES Physician Database to identify prescriber specialties.

### 2.3. Cohort Description

We identified all medications with an indication for cancer treatment reimbursed through the ODB program and dispensed from a community pharmacy (i.e., take-home cancer medications) between 1 April 2017 and 31 March 2021. See Supplementary Table S1 for a complete list of medications included in our study. If the dispensed medication could be prescribed for both cancer and non-cancer-related indications (e.g., methotrexate), we only included claims in which the recipient met our definition of a cancer diagnosis. Specifically, we used a health administrative claims algorithm to identify individuals with active cancer, defined as the presence of at least of the following criteria: (1) medication prescribed by a physician with a cancer specialty; (2) clinic visit for systemic therapy within five days of the medication dispense date; (3) cancer diagnosis date in the Ontario Cancer Registry in the 182 days prior to the medication dispense date; or (4) record for cancer treatment (e.g., chemotherapy, radiation therapy, or cancer surgery) in the 182 days prior to the medication dispense date. See Supplementary Table S2 for codes used to define active cancer. Lastly, we excluded all claims dispensed to individuals without a valid OHIP number or with missing age or sex information in the RPDB as well as claims for medications indicated for supportive therapy (e.g., anti-nausea medications) and claims for those who died prior to the prescription dispense date. For all remaining claims, no missing data was observed, and therefore no handling of missing data was required.

### 2.4. Outcomes

Our primary outcome was per-beneficiary spending on take-home cancer medications reimbursed by the ODB program. The denominator was defined as the number of unique ODB beneficiaries who were dispensed at least one cancer-indicated medication. For the numerator, we defined total ODB spending on take-home cancer medications, which included the dollar amount reimbursed by ODB toward medication costs and any associated pharmacist dispensing fees. As secondary outcomes, we determined total ODB spending on take-home cancer medications and the number of beneficiaries who were dispensed one or more take-home cancer medication(s). All outcomes were reported overall and stratified by drug class (i.e., small-molecule targeted therapies, cytotoxic chemotherapies, immunotherapies, hormonal therapies).

### 2.5. Statistical Analysis

We reported our primary outcome by fiscal year and month to visualize trends over the study period, reported as nominal cost in Canadian dollars. For each drug class, we calculated its contribution to the total growth in ODB spending over the study period by dividing the difference in ODB spending on the drug class of interest by the overall difference in ODB spending between fiscal years 2017/18 and 2020/21. We used autoregressive integrated moving average (ARIMA) models to evaluate the impact of the declaration of emergency for SARS-CoV-2 on monthly trends for the primary and secondary outcomes, overall and across all stratifications. We defined the month of March 2020 as the intervention month for all ARIMA models, as it contains the date (17 March 2020) that the state of emergency for SARS-CoV-2 was first declared in Ontario. We hypothesized that the SARS-CoV-2 intervention would lead to a temporary increase in the reimbursement of cancer-indicated medications through the ODB program (e.g., provision of take-home cancer medications), along with a gradual increase over time. Therefore, we used pulse and ramp transfer functions to test for temporary (between March and June 2020) and gradual changes in the trend, respectively. The Dickey–Fuller test and the Ljung–Box test were used to assess for stationary, seasonality, and white noise for ARIMA models. For the selection of model parameters (i.e., p/q terms), we assessed autocorrelation, partial autocorrelation, and inverse autocorrelation correlograms. A type 1 error of 5% was chosen to evaluate statistical significance. We conducted all analyses at ICES using SAS Enterprise Guide Version 7.15 and SAS OnDemand for Academics (https://www.sas.com/en_ca/software/on-demand-for-academics.html (accessed on 15 April 2025), SAS Institute, Cary, NC, USA).

## 3. Results

### 3.1. Trends per Fiscal Year

Between fiscal year 2017/18 and 2020/21, public payer spending on take-home cancer medicines increased by 32.8%, from CAD 4422 to CAD 6579 per-beneficiary (Table 1). When stratified by drug class, annual per-beneficiary spending was highest among recipients of medications from the immunotherapy (CAD 72,544 per beneficiary in FY 2020/21) and small-molecule targeted therapy (CAD 44,544 per beneficiary in FY 2020/21) drug classes. Per-beneficiary spending on the hormonal therapy and cytotoxic chemotherapy drug classes was much lower in comparison and remained relatively stable over time, with averages of CAD 3154 and CAD 275 spent per recipient in FY 2020/21, respectively.

The rise in per-beneficiary spending was primarily driven by the rising cost of take-home cancer medications, which outpaced the rise in the number of ODB beneficiaries who were dispensed these medications. Specifically, despite a 68.5% increase in ODB expenditures on take-home cancer medications (from CAD 455,860,978 in fiscal year 2017/18 to CAD 768,016,588 in fiscal year 2020/21), the number of ODB beneficiaries increased by only 13.3% (N = 103,085 to N = 116,744). When stratified by drug class, public payer expenditure on small-molecule targeted therapies increased by 127.5% (from CAD 164,268,141 to CAD 373,631,344) and accounted for approximately two-thirds (67.1%) of the total increase in take-home cancer medication expenditures between 2017/18 and 2020/21. Similarly, spending on medications from the immunotherapy drug class rose by 50.1%, from CAD 141,834,933 to CAD 212,915,798, and accounted for 22.8% of the increase in public payer spending on take-home cancer treatments. Although public payer expenditures on small-molecule targeted therapies and immunotherapy accounted for nearly three-quarters of the total program spending in FY 2020/21, these medications were dispensed to 7.2% (N = 8388) and 2.5% (N = 2935) of beneficiaries, respectively. In contrast, public payer spending on cytotoxic chemotherapy and hormonal therapy accounted for 2.0% (CAD 15,502,283) and 21.6% (CAD 165,967,163) of the overall spending on take-home cancer medications, while being dispensed to 48.3% (N = 56,041) and 45.1% (N = 52,625) of ODB beneficiaries in FY 2020/21.

### 3.2. Monthly Trends and the Impact of SARS-COV-2

**Figure 1:**
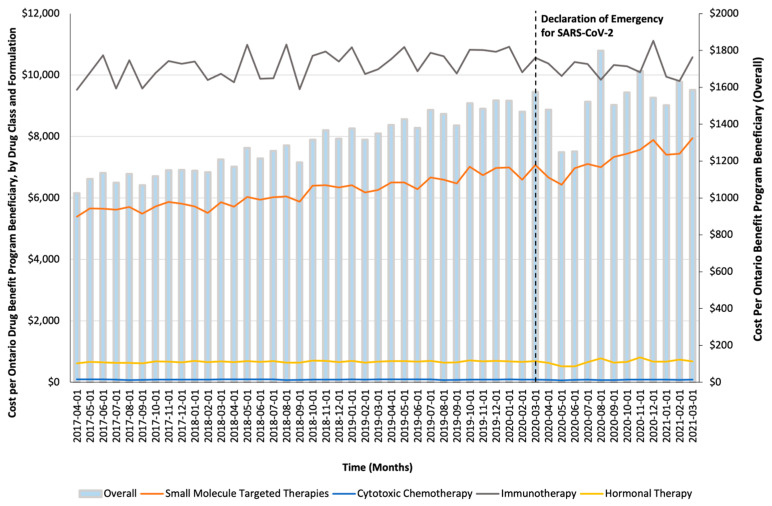
Trends in the per-beneficiary Ontario Drug Benefit program’s spending on take-home oncology medications before and after the declaration of emergency for SARS-CoV-2, reported overall and stratified by drug class.

**Figure 2:**
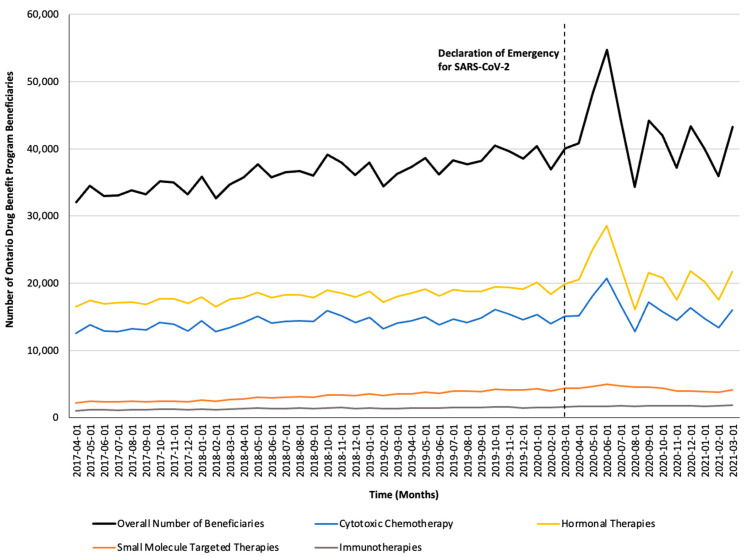
Trends in the number of Ontario Drug Benefit Program beneficiaries who were dispensed take-home oncology medication before and after the declaration of emergency for SARS-CoV-2, reported overall and stratified by drug class.

**Figure 3:**
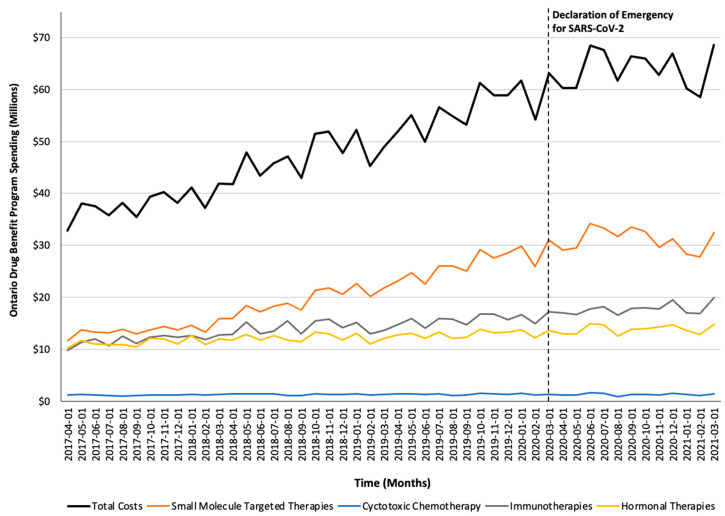
Trends in the Ontario Drug Benefit program’s spending on take-home oncology medications before and after the declaration of emergency for SARS-CoV-2, reported overall and stratified by drug class.

Monthly trends for per-beneficiary spending are depicted in Figure 1. Between April 2017 and February 2020, per-beneficiary expenditures on take-home cancer medications steadily increased. However, upon declaration of emergency for SARS-CoV-2 in March 2020 (i.e., intervention month), a short-term yet significant decline in per-beneficiary expenditures between March and June 2020 was noted (CAD −181 per month; 95% confidence interval [CI]: CAD −264, CAD −98; *p*-value < 0.01; Table 2). In April 2020, per-beneficiary spending declined to CAD 1468 (from CAD 1527 in March 2020), reaching CAD 1252 in June 2020, returning to the trend observed prior to the SARS-CoV-2 intervention a month thereafter. The short-term decline in per-beneficiary spending was driven by a significant and temporary increase in ODB beneficiaries who were dispensed low-cost medicines from the hormonal and cytotoxic drug classes (Figure 2) without a corresponding rise in public payer spending (Figure 3). Specifically, between March 2020 and June 2020, the number of ODB beneficiaries who were dispensed cancer medication increased by 5670 individuals per month (95% CI: 2254, 9086; *p*-value < 0.01), with the trend returning to numbers observed prior to the intervention month in August 2020. When stratified by drug class, the number of individuals who were dispensed medications from the hormonal and cytotoxic chemotherapy drug classes increased by 3605 (95% CI: 1972–5237; *p* < 0.01) and 2258 (95% CI: 795–3721; *p* < 0.01) per month, respectively, between March and June 2020. In contrast, the SARS-CoV-2 intervention did not impact the number of beneficiaries who were dispensed high-cost medications from the immunotherapy drug class and only minimally increased the number of beneficiaries who were dispensed medications from the small-molecule targeted therapy drug class (+265 per month; 95% CI: 13, 518; *p*-value = 0.04). Despite the transient increase in ODB beneficiaries, the rising trend of ODB expenditures on take-home cancer medications significantly slowed following the declaration of emergency for SARS-CoV-2 (ramp transfer function estimate: −763,046; 95% CI: −1,341,102, −184,991; *p*-value < 0.01) and reached a plateau for the reminder of the study period. When stratified by drug class, no changes in expenditures following the SARS-CoV-2 intervention were noted.

## 4. Discussion

In this repeated cross-sectional population-based study examining trends in the uptake and cost of publicly funded take-home cancer medications in Ontario, Canada, we observed a 32.8% increase in per-beneficiary spending on take-home cancer medications between FY 2017/18 and 2020/21. Our results highlight the fact that this rise in per-beneficiary spending was largely driven by the increased costs of medications within the small-molecule targeted therapy and immunotherapy drug classes, which accounted for 48.6% (CAD 373 million) and 27.7% (CAD 212 million) of the total program spending in fiscal year 2020/21, respectively. Despite making up nearly three-quarters of public payer spending in this area, the uptake of these medications remained low as less than 8% of the ODB beneficiaries included in our study population who were dispensed medications from the aforementioned drug categories. Lastly, the declaration of emergency for SARS-CoV-2 led to a short-term yet significant increase in the number of beneficiaries who were dispensed low-cost take-home cancer medications from the cytotoxic chemotherapy and hormonal therapy drug classes, which likely reflects the pandemic-related transitions from in-hospital to community-based treatment.

Prior research conducted by Del Paggio et al. examined trends in the costs of all publicly reimbursed cancer-indicated medications in Ontario, Canada, including those administered in-person and those dispensed as take-home treatment [10]. Their findings reported an average annual increase of 15% in public payer expenditures on cancer-indicated medications between 2012 and 2022, with an acceleration in spending over the past four years [10]. We build upon findings reported by Del Paggio et al. as we considered trends in public payer reimbursement of take-home cancer medications across different drug classes and the impact of SARS-CoV-2 on medication uptake among people who met our definition of an active cancer diagnosis. When focusing solely on take-home cancer treatment, we noted a similar annual increase of 14% between fiscal year 2017/18 and 2020/21. Meanwhile, overall public payer spending on prescription medication across Canada increased by an average of 4% per annum between 2017 [13] (CAD 13.5 billion) and 2021 [14] (CAD 16.2 billion), highlighting the disproportionate increase in the cost of cancer medicines compared to the broader pharmaceutical market.

We found that the declaration of emergency for SARS-CoV-2 and accompanying reallocation of in-patient healthcare resources towards pandemic management [9] was associated with a temporary shift towards use of low-cost take-home cancer medications. The uptake of high-cost medication from the immunotherapy and small-molecule targeted therapy drug class generally remained low over our study period and was unchanged by the declaration of emergency for SARS-CoV-2. Access to high-cost take-home cancer treatments may be limited due to requirements for special authorization prior to medication reimbursement by the public payer and the overall fragmented nature of public drug coverage for those not eligible for the ODB program [6,7,11]. Individuals without public drug coverage in Ontario must exhaust any available household insurance and spend a designated amount out-of-pocket on eligible prescription drugs—calculated as a deductible equivalent to 4% of household income—before they receive reimbursement through ODB [15]. When compared to Canadian provinces with comprehensive cancer-treatment public drug coverage (i.e., take-home and hospital-administered medications), the uptake of take-home cancer medications was 40% lower among Ontario residents who were not insured by the ODB program [16]. Calls have been made for Ontario to consider a centralized cancer care model to reduce the financial and administrative barriers to take-home cancer treatment and allow for seamless and timely provision of treatment as prescribed regardless of mode of delivery [6,16,7]. Future research should examine whether the extra administrative requirements for special authorization and Trillium program eligibility has deterred Ontario residents from receiving take-home cancer medications, particularly during periods of public health emergencies such as SARS-CoV-2 when resources are limited.

A core strength of our study is its use of a large, population-based database which captures publicly reimbursed medication across the province. However, our study has some limitations that warrant further discussion. First, we may have overestimated public payer spending on take-home cancer medications as we relied on the listing prices for all our calculations, which did not include manufacturers’ rebates received by the public payer. Second, medications with multiple indications (e.g., methotrexate) may have been misclassified as a take-home cancer medication. However, we expect this number to be relatively small as we restricted our analysis to those individuals who met our definition of having active cancer. Third, we estimated the utilization of take-home cancer medications based on the number of unique beneficiaries who were dispensed cancer-indicated medication. We did not estimate dispensing volume and therefore cannot determine whether the prescribing patterns remained consistent following the declaration of emergency for SARS-CoV-2. Fourth, causal inferences cannot be established based on our ARIMA models due to the possibility of additional events occurring in close proximity to the declaration of emergency for SARS-CoV-2. Lastly, the trends observed in utilization and spending may not be generalizable to other jurisdictions due to differences in the public payer reimbursement of healthcare services.

## 5. Conclusions

Between 2017 and 2021, we observed a 32.8% rise in per-beneficiary spending on take-home cancer medications in Ontario, Canada. The rise in public payer spending was primarily attributed to the reimbursement of higher-cost medications from the small-molecule targeted therapy and immunotherapy drug classes despite being dispensed to less than 10% of the study population. Although the declaration of emergency for SARS-CoV-2 resulted in a temporary increase in the number of ODB beneficiaries who were dispensed take-home cancer medication, no corresponding rise in public payer spending was noted. Future research should further investigate whether increased administrative requirements for special authorization approvals or eligibility requirements for the ODB program has contributed to limited access to take-home cancer medication in Ontario, Canada, particularly during periods of public health emergency.

## Figures and Tables

**Table 1:** Annual expenditure on take-home oncology medications by the Ontario Drug Benefit Program, stratified by drug class and route of administration from 1 April 2017 to 31 March 2021 (nominal cost unadjusted).

Oncology Medications	2017/18			2018/19			2019/20			2020/21			2017–2021
	No. Beneficiaries (% Overall)	Total Program Spending (% Overall)	Per-Beneficiary Spending	No. Beneficiaries (% Overall)	Total Program Spending (% Overall)	Per-Beneficiary Spending	No. Beneficiaries (% Overall)	Total Program Spending (% Overall)	Per-Beneficiary Spending	No. Beneficiaries (%)	Total Program Spending (% Overall)	Per-Beneficiary Spending	Percent Contribution to Total Growth
Overall	103,085	$455,860,978	$ 4422	110,483	$566,631,775	$5129	114,982	$679,795,654	$5912	116,744	$768,016,588	$6579	-
**Drug Class**													
*Small-Molecule Targeted Therapies*	5232 (5.1%)	$164,268,141 (36.0)	$31,397	6486 (5.9)	$234,562,652 (41.4)	$36,164	7677 (6.7)	$319,479,265 (47.0)	$41,615	8388 (7.2)	$373,631,344 (48.6)	$44,544	67.1
*Cytotoxic Chemotherapy*	51,146 (49.6%)	$14,244,528 (3.1)	$279	55,525 (50.3)	$15,527,400 (2.7)	$280	57,577 (50.1)	$15,941,767 (2.3)	$277	56,041(48.3)	$15,502,283 (2.0)	$275	0.4
*Immuno-therapy*	2118(2.1%)	$141,834,933 (31.1)	$66,966	2407(2.2)	$170,039,129 (30.0)	$70,644	2691(2.3)	$188,953,933 (27.8)	$70,217	2935(2.5)	$212,915,798 (27.7)	$72,544	22.8
*Hormonal Therapy*	46,743 (45.3%)	$135,513,377 (29.7)	$2899	49,077(44.4)	$146,502,594 (25.9)	$2985	50,064(44.0)	$155,420,689 (22.9)	$3071	52,625(45.1)	$165,967,163 (21.6)	$3154	9.8
**Formulation**													
*Oral*	72,914 (70.7%)	$367,788,358 (80.7)	$5044	77,149(69.8)	$471,722,645 (83.3)	$6114	79,886(69.5)	$579,001,389 (85.2)	$7248	82,184(70.4)	$662,192,365 (86.2)	$8057	94.3
*Parenteral*	19,556 (19.0%)	$87,324,011 (19.2)	$4465	20,331(18.4)	$94,061,822 (16.6)	$4627	19,770(17.2)	$99,848,387 (14.7)	$5051	19,670(16.8)	$104,935,138 (13.7)	$5335	5.6

**Table 2:** Autoregressive interventional moving average (ARIMA) models’ results, examining the impact of the SARS-COV-2 pandemic on the monthly utilization of and cost trends for take-home cancer treatments dispensed through the Ontario Drug Benefits Program (ODB).

Outcome of Interest	Model Parameters	Transfer Function	Estimate (95% CI)	*p*-Value
**ODB Spending Per-Beneficiary**				
Overall	(0,1,4)	Pulse	**−181 (−264, −98)**	**<0.01**
		Ramp	−5 (−24, 14)	0.64
Small-Molecule Targeted Therapies	(3,1,0)	Pulse	−168 (−434, 99)	0.22
		Ramp	64 (−11, 139)	0.09
Cytotoxic Chemotherapy	(3,1,0) × (0,1,0)_12_	Pulse	**−13 (−18, −8)**	**<0.01**
		Ramp	−1 (−2, 0)	0.16
Immunotherapy	(2,1,0) × (0,1,0)_12_	Pulse	−195 (−396, 7)	0.06
		Ramp	**−57 (−112, −3)**	**0.04**
Hormonal Therapy	Did not pass white noise test			
**Total ODB Spending**				
Overall	(2,1,0) × (0,1,0)_12_	Pulse	−906,816 (−3,025,327, 1,211,696)	0.40
		Ramp	**−763,046 (−1,341,102, −184,991)**	**<0.01**
Small-Molecule Targeted Therapies	(3,1,0)	Pulse	291,846 (−1,769,887, 2,353,579)	0.78
		Ramp	223,000 (−658,349, 1,104,348)	0.62
Cytotoxic Chemotherapy	(4,1,0) × (0,1,0)_12_	Pulse	−78,745 (−188,431, 30,942)	0.16
		Ramp	−12,765 (−36,459, 10,928)	0.29
Immunotherapy	(2,1,0) × (0,1,0)_12_	Pulse	341,122 (−269,770, 952,014)	0.27
		Ramp	−31,037 (−195,496, 133,421)	0.71
Hormonal Therapy	(2,1,0) × (0,1,0)_12_	Pulse	−415,646 (−961,530, 130,238)	0.14
		Ramp	−62,715 (−214,976, 89,546)	0.42
**Number of ODB Beneficiaries**				
Overall	(5,1,0)	**Pulse**	**5670 (2254, 9086)**	**<0.01**
		Ramp	176 (−393, 744)	0.54
Small-Molecule Targeted Therapies	(5,1,0)	**Pulse**	**265 (13, 518)**	**0.04**
		Ramp	19 (−83, 122)	0.71
Cytotoxic Chemotherapy	(0,1,3)	**Pulse**	**2258 (795, 3721)**	**<0.01**
		Ramp	61 (−122, 245)	0.51
Immunotherapy	(3,1,0) × (0,1,0)_12_	Pulse	26 (−26, 78)	0.33
		Ramp	−1 (−26, 23)	0.91
Hormonal Therapy	(5,1,0)	**Pulse**	**3605 (1972, 5237)**	**<0.01**
		Ramp	107 (−157, 369)	0.43

## Data Availability

The datasets presented in this article are not readily available because they are held securely in coded form at ICES. While legal data-sharing agreements between ICES and data providers (e.g., healthcare organizations and government) prohibit ICES from making the dataset publicly available, access may be granted to those who meet prespecified criteria for confidential access, available at https://www.ices.on.ca/use-ices-data/ (accessed on 22 March 2022).

## Supplementary Materials

The following are available online at https://www.mdpi.com/article/10.3390/curroncol32040237/s1: Table S1: Drug Identification Numbers for Included Medications and Table S2: Active Cancer Diagnosis Definition Codes.
