# Supplementary material for: Trends in the Cost and Utilization of Publicly Reimbursed Cancer Medications Dispensed as Take-Home Treatments from 2017–2021
Source: Curr Oncol. 2025 Apr 18;32(4):237. doi: 10.3390/curroncol32040237 (PMC12025805; doi:10.3390/curroncol32040237)
Supplement: Supplementary file 1 [file curroncol-32-00237-s001.zip › curroncol-3554164-supplementary.pdf]

Supplementary Table S1. Drug Identification Numbers for Included Medications

Drug Identification Number	Brand Name	Generic Name
02371065	Zytiga	ABIRATERONE ACETATE
02457113	Zytiga	ABIRATERONE ACETATE
02415666	Giotrif	AFATINIB
02415674	Giotrif	AFATINIB
02415682	Giotrif	AFATINIB
02458136	Alecensaro	ALECTINIB HYDROCHLORIDE
02145839	Vesanoid	ALL-TRANS RETINOIC ACID
02126230	Hexalen	ALTRETAMINE
02260107	Sandoz Anagrelide	ANAGRELIDE HCL
02236859	Agrylin	ANAGRELIDE HCL
02236860	Agrylin	ANAGRELIDE HCL
02224135	Arimidex	ANASTROZOLE
02313049	Teva-Anastrozole	ANASTROZOLE
02320738	PMS-Anastrozole	ANASTROZOLE
02326035	Zinda-Anastrozole	ANASTROZOLE
02328690	Ran-Anastrozole	ANASTROZOLE
02338467	Sandoz Anastrozole	ANASTROZOLE
02339080	Jamp-Anastrozole	ANASTROZOLE
02351218	Anastrozole	ANASTROZOLE
02361418	Mylan-Anastrozole	ANASTROZOLE
02365650	Taro-Anastrozole	ANASTROZOLE
02374420	Apo-Anastrozole	ANASTROZOLE
02379104	Med-Anastrozole	ANASTROZOLE
02379562	Mar-Anastrozole	ANASTROZOLE
02393573	Mint-Anastrozole	ANASTROZOLE
02394898	Co Anastrozole	ANASTROZOLE
02404990	Auro-Anastrozole	ANASTROZOLE
02417855	Nat-Anastrozole	ANASTROZOLE
02427818	Van-Anastrozole	ANASTROZOLE
02458799	CCP-Anastrozole	ANASTROZOLE
02478374	Erleada	APALUTAMIDE
02389630	Inlyta	AXITINIB
02389649	Inlyta	AXITINIB
02184478	Casodex	BICALUTAMIDE
02270226	Teva-Bicalutamide	BICALUTAMIDE
02274337	Co Bicalutamide	BICALUTAMIDE
02275589	PMS-Bicalutamide	BICALUTAMIDE
02276089	Sandoz Bicalutamide	BICALUTAMIDE
02277700	Ratio-Bicalutamide	BICALUTAMIDE

02296063	Apo-Bicalutamide	BICALUTAMIDE
02302403	Mylan-Bicalutamide	BICALUTAMIDE
02325985	Bicalutamide Tablets	BICALUTAMIDE
02357216	Jamp-Bicalutamide	BICALUTAMIDE
02371324	Ran-Bicalutamide	BICALUTAMIDE
02428709	Van-Bicalutamide	BICALUTAMIDE
02419149	Bosulif	BOSUTINIB
02419157	Bosulif	BOSUTINIB
02225158	Suprefact	BUSERELIN ACETATE
02225166	Suprefact	BUSERELIN ACETATE
02228955	Suprefact Depot	BUSERELIN ACETATE
02240749	Suprefact Depot	BUSERELIN ACETATE
00004618	Myleran	BUSULFAN
02480824	Cabometyx	CABOZANTINIB
02480832	Cabometyx	CABOZANTINIB
02480840	Cabometyx	CABOZANTINIB
02238453	Xeloda	CAPECITABINE
02238454	Xeloda	CAPECITABINE
02400022	Teva-Capecitabine	CAPECITABINE
02400030	Teva-Capecitabine	CAPECITABINE
02421917	Sandoz Capecitabine	CAPECITABINE
02421925	Sandoz Capecitabine	CAPECITABINE
02426757	Ach-Capecitabine	CAPECITABINE
02426765	Ach-Capecitabine	CAPECITABINE
02457490	Taro-Capecitabine	CAPECITABINE
02457504	Taro-Capecitabine	CAPECITABINE
02436779	Zykadia	CERITINIB
00004626	Leukeran	CHLORAMBUCIL
02452340	Cotellic	COBIMETINIB FUMARATE
02384256	Xalkori	CRIZOTINIB
02384264	Xalkori	CRIZOTINIB
00013544	Procytox	CYCLOPHOSPHAMIDE
00013552	Procytox	CYCLOPHOSPHAMIDE
00013749	Procytox	CYCLOPHOSPHAMIDE
00262676	Procytox	CYCLOPHOSPHAMIDE
00344877	Cytoxan	CYCLOPHOSPHAMIDE
00344885	Cytoxan	CYCLOPHOSPHAMIDE
00344915	Cytoxan	CYCLOPHOSPHAMIDE
02241795	Procytox	CYCLOPHOSPHAMIDE
02241796	Procytox	CYCLOPHOSPHAMIDE
02241797	Procytox	CYCLOPHOSPHAMIDE

02241799	Procytox	CYCLOPHOSPHAMIDE
02241800	Procytox 2000mg/Vial Injection	CYCLOPHOSPHAMIDE
02241798	Procytox	CYCLOPHOSPHAMIDE INJECTION
00704423	Androcur Depot	CYPROTERONE ACETATE
00704431	Androcur	CYPROTERONE ACETATE
02229449	Alti-CPA	CYPROTERONE ACETATE
02229723	Gen-Cyproterone	CYPROTERONE ACETATE
02232872	Novo-Cyproterone	CYPROTERONE ACETATE
02245898	Cyproterone	CYPROTERONE ACETATE
02390760	Med-Cyproterone	CYPROTERONE ACETATE
02409607	Tafinlar	DABRAFENIB
02409615	Tafinlar	DABRAFENIB
02496348	Nubeqa	DAROLUTAMIDE
02293129	Sprycel	DASATINIB
02293137	Sprycel	DASATINIB
02293145	Sprycel	DASATINIB
02320193	Sprycel	DASATINIB
02470705	Apo-Dasatinib	DASATINIB
02470713	Apo-Dasatinib	DASATINIB
02470721	Apo-Dasatinib	DASATINIB
02481499	Apo-Dasatinib	DASATINIB
01926683	Cerubidine	DAUNORUBICIN
02337029	Firmagon	DEGARELIX ACETATE
02337037	Firmagon	DEGARELIX ACETATE
02407329	Xtandi	ENZALUTAMIDE
02269007	Tarceva	ERLOTINIB
02269015	Tarceva	ERLOTINIB
02269023	Tarceva	ERLOTINIB
02377691	Teva-Erlotinib	ERLOTINIB
02377705	Teva-Erlotinib	ERLOTINIB
02377713	Teva-Erlotinib	ERLOTINIB
02454386	PMS-Erlotinib	ERLOTINIB
02454394	PMS-Erlotinib	ERLOTINIB
02461862	Apo-Erlotinib	ERLOTINIB
02461870	Apo-Erlotinib	ERLOTINIB
02461889	Apo-Erlotinib	ERLOTINIB
02483912	Nat-Erlotinib	ERLOTINIB
02483920	Nat-Erlotinib	ERLOTINIB
02483939	Nat-Erlotinib	ERLOTINIB
00780278	Emcyt	ESTRAMUSTINE PHOSPHATE DISODIUM

02063794	Emcyt	ESTRAMUSTINE PHOSPHATE DISODIUM
02080036	Etoposide	ETOPOSIDE
00616192	Vepesid	ETOPOSIDE
02241182	Etoposide Injection USP	ETOPOSIDE
02380935	Etoposide Injection USP	ETOPOSIDE
02339501	Afinitor	EVEROLIMUS
02339528	Afinitor	EVEROLIMUS
02369257	Afinitor	EVEROLIMUS
02425645	Afinitor Disperz	EVEROLIMUS
02425653	Afinitor Disperz	EVEROLIMUS
02425661	Afinitor Disperz	EVEROLIMUS
02450267	Afinitor	EVEROLIMUS
02463229	Teva-Everolimus	EVEROLIMUS
02463237	Teva-Everolimus	EVEROLIMUS
02463253	Teva-Everolimus	EVEROLIMUS
02242705	Aromasin	EXEMESTANE
02390183	Co Exemestane	EXEMESTANE
02407841	Med-Exemestane	EXEMESTANE
02408473	Teva-Exemestane	EXEMESTANE
02419726	Apo-Exemestane	EXEMESTANE
02246226	Fludara	FLUDARABINE PHOSPHATE
09854515	Fludarabine	FLUDARABINE PHOSPHATE
00012882	Fluorouracil	FLUOROURACIL
00330582	Efudex	FLUOROURACIL
00637726	Euflex	FLUTAMIDE
02230089	Teva-Flutamide	FLUTAMIDE
02230104	PMS-Flutamide	FLUTAMIDE
02238560	Apo-Flutamide	FLUTAMIDE
02248624	Faslodex	FULVESTRANT
02460130	Teva-fulvestrant	FULVESTRANT
02483610	Teva-fulvestrant	FULVESTRANT
02248676	Iressa	GEFITINIB
02468050	Apo-Gefitinib	GEFITINIB
02487748	Sandoz Gefitinib	GEFITINIB
02491796	Nat-Gefitinib	GEFITINIB
00857599	ZOLADEX	GOSERELIN ACETATE
02049325	Zoladex	GOSERELIN ACETATE
02225905	Zoladex LA	GOSERELIN ACETATE
00465283	Hydrea	HYDROXYUREA
02242920	Mylan-Hydroxyurea	HYDROXYUREA
02247937	Apo-Hydroxyurea	HYDROXYUREA

02343096	Hydroxyurea	HYDROXYUREA
02434407	Imbruvica	IBRUTINIB
02438798	Zydelig	IDELALISIB
02438801	Zydelig	IDELALISIB
02244725	Gleevec	IMATINIB MESYLATE
02253275	Gleevec	IMATINIB MESYLATE
02253283	Gleevec	IMATINIB MESYLATE
02355337	Apo-Imatinib	IMATINIB MESYLATE
02355345	Apo-Imatinib	IMATINIB MESYLATE
02397285	Nat-Imatinib	IMATINIB MESYLATE
02397293	Nat-Imatinib	IMATINIB MESYLATE
02399806	Teva-Imatinib	IMATINIB MESYLATE
02399814	Teva-Imatinib	IMATINIB MESYLATE
02431114	PMS-Imatinib	IMATINIB MESYLATE
02431122	PMS-Imatinib	IMATINIB MESYLATE
09857533	PMS-Imatinib	IMATINIB MESYLATE
09857534	PMS-Imatinib	IMATINIB MESYLATE
09857444	Apo-Imatinib	IMATINIB MESYLATE
09857446	Apo-Imatinib	IMATINIB MESYLATE
09857447	Gleevec	IMATINIB MESYLATE
09857448	Gleevec	IMATINIB MESYLATE
09857449	Teva-Imatinib	IMATINIB MESYLATE
09857450	Teva-Imatinib	IMATINIB MESYLATE
09857468	Nat-Imatinib	IMATINIB MESYLATE
09857469	Nat-Imatinib	IMATINIB MESYLATE
02239505	Aldara	IMIQUIMOD
02407825	Apo-Imiquimod	IMIQUIMOD
00705896	INTRON A	INTERFERON ALFA-2B
00705918	INTRON A	INTERFERON ALFA-2B
00705926	INTRON A	INTERFERON ALFA-2B
00889067	INTRON A	INTERFERON ALFA-2B
02223384	Intron A	INTERFERON ALFA-2B
02223392	Intron A	INTERFERON ALFA-2B
02223406	Intron A	INTERFERON ALFA-2B
02223414	Intron A	INTERFERON ALFA-2B
02231651	Intron A	INTERFERON ALFA-2B
02238674	Intron A	INTERFERON ALFA-2B
02238675	Intron A	INTERFERON ALFA-2B
02240693	Intron A	INTERFERON ALFA-2B
02240694	Intron A	INTERFERON ALFA-2B
02240695	Intron A	INTERFERON ALFA-2B

09853995	Intron A	INTERFERON ALFA-2B
09854045	Intron A	INTERFERON ALFA-2B
09854053	Intron A	INTERFERON ALFA-2B
02283395	Somatuline Autogel	LANREOTIDE ACETATE
02283409	Somatuline Autogel	LANREOTIDE ACETATE
02283417	Somatuline Autogel	LANREOTIDE ACETATE
02326442	Tykerb	LAPATINIB
02304899	Revlimid	LENALIDOMIDE
02304902	Revlimid	LENALIDOMIDE
02317699	Revlimid	LENALIDOMIDE
02317710	Revlimid	LENALIDOMIDE
02440601	Revlimid	LENALIDOMIDE
02459418	Revlimid	LENALIDOMIDE
02450291	Lenvima	LENVATINIB
02450305	Lenvima	LENVATINIB
02450313	Lenvima	LENVATINIB
02450321	Lenvima	LENVATINIB
02468220	Lenvima	LENVATINIB
02484056	Lenvima	LENVATINIB
02484129	Lenvima	LENVATINIB
02231384	Femara	LETROZOLE
02309114	PMS-Letrozole	LETROZOLE
02322315	Med-Letrozole	LETROZOLE
02338459	Letrozole Tablets USP	LETROZOLE
02343657	Teva-Letrozole	LETROZOLE
02344815	Sandoz Letrozole	LETROZOLE
02347997	Letrozole	LETROZOLE
02348969	Letrozole	LETROZOLE
02358514	Apo-Letrozole	LETROZOLE
02372169	Myl-Letrozole	LETROZOLE
02372282	Ran-Letrozole	LETROZOLE
02373009	Jamp-Letrozole	LETROZOLE
02373424	Mar-Letrozole	LETROZOLE
02378213	Zinda-Letrozole	LETROZOLE
02404400	Auro-Letrozole	LETROZOLE
02421585	Nat-Letrozole	LETROZOLE
02428156	Van-Letrozole	LETROZOLE
02459884	CCP-Letrozole	LETROZOLE
00727695	LUPRON	LEUPROLIDE ACETATE
00836273	Lupron Depot PDS	LEUPROLIDE ACETATE
00884502	Lupron Depot PDS	LEUPROLIDE ACETATE

02230248	Lupron Depot PDS	LEUPROLIDE ACETATE
02239833	Lupron Depot PDS	LEUPROLIDE ACETATE
02239834	Lupron Depot PDS	LEUPROLIDE ACETATE
02248239	Eligard	LEUPROLIDE ACETATE
02248240	Eligard	LEUPROLIDE ACETATE
02248999	Eligard	LEUPROLIDE ACETATE
02268892	Eligard	LEUPROLIDE ACETATE
00360414	CeeNU	LOMUSTINE (CCNU)
00360422	CeeNU	LOMUSTINE (CCNU)
00360430	CeeNU	LOMUSTINE (CCNU)
02176092	Lin-Megestrol	MEGESTROL ACETATE
02176106	Lin-Megestrol	MEGESTROL ACETATE
02195917	Megestrol	MEGESTROL ACETATE
02195925	Megestrol	MEGESTROL ACETATE
00004715	Alkeran	MELPHALAN
00004723	Purinethol	MERCAPTOPURINE
02415275	Mercaptopurine Tablets USP	MERCAPTOPURINE
09857458	Purinethol	MERCAPTOPURINE
00014915	Methotrexate	METHOTREXATE
00321397	Methotrexate	METHOTREXATE
00614343	Methotrexate	METHOTREXATE
00874132	Rheumatrex	METHOTREXATE
02099705	Methotrexate Sodium Injection	METHOTREXATE
02170663	Methotrexate (No Preservative)	METHOTREXATE
02170671	Methotrexate	METHOTREXATE
02170698	PMS-Methotrexate	METHOTREXATE
02171767	Rheumatrex	METHOTREXATE
02182750	Methotrexate	METHOTREXATE
02182777	Methotrexate	METHOTREXATE
02182947	Methotrexate Sodium	METHOTREXATE
02182955	Methotrexate Injection USP	METHOTREXATE
02182963	Apo-Methotrexate	METHOTREXATE
02244798	Ratio-Methotrexate Sodium	METHOTREXATE
02398427	Methotrexate Injection USP	METHOTREXATE
02417626	Methotrexate Injection, USP	METHOTREXATE
09857520	Methotrexate	METHOTREXATE
02466236	Rydapt	MIDOSTAURIN
02315874	Tasigna	NILOTINIB
02368250	Tasigna	NILOTINIB
00863890	ANANDRON	NILUTAMIDE
00863904	ANANDRON	NILUTAMIDE

01989642	ANANDRON	NILUTAMIDE
01989650	ANANDRON	NILUTAMIDE
02221861	Anandron	NILUTAMIDE
02221888	Anandron	NILUTAMIDE
09850635	Nitrogen mustard	NITROGEN MUSTARD
00839191	Sandostatin	OCTREOTIDE
00839205	Sandostatin	OCTREOTIDE
00839213	Sandostatin	OCTREOTIDE
02049392	Sandostatin	OCTREOTIDE
02239323	Sandostatin LAR	OCTREOTIDE
02239324	Sandostatin LAR	OCTREOTIDE
02239325	Sandostatin LAR	OCTREOTIDE
02248639	Octreotide Acetate Omega	OCTREOTIDE
02248640	Octreotide Acetate Omega	OCTREOTIDE
02248641	Octreotide Acetate Omega	OCTREOTIDE
02248642	Octreotide Acetate Omega	OCTREOTIDE
09854528	Octreotide	OCTREOTIDE
09854540	Sandostatin	OCTREOTIDE
09857228	Sandostatin	OCTREOTIDE
09857229	Sandostatin	OCTREOTIDE
09857231	Sandostatin	OCTREOTIDE
02454408	Lynparza	OLAPARIB
02475200	Lynparza	OLAPARIB
02475219	Lynparza	OLAPARIB
02456214	Tagrisso	OSIMERTINIB MESYLATE
02456222	Tagrisso	OSIMERTINIB MESYLATE
02453150	Ibrance	PALBOCICLIB
02453169	Ibrance	PALBOCICLIB
02453177	Ibrance	PALBOCICLIB
02352303	Votrient	PAZOPANIB
02248077	Pegasys	PEGINTERFERON ALFA 2A RECOMBINANT
09857505	Pegasys	PEGINTERFERON ALFA 2A RECOMBINANT
02419580	Pomalyst	POMALIDOMIDE
02419599	Pomalyst	POMALIDOMIDE
02419602	Pomalyst	POMALIDOMIDE
02419610	Pomalyst	POMALIDOMIDE
02437333	Iclusig	PONATINIB
02437341	Iclusig	PONATINIB
02019876	Photofrin PWS	PORFIMER SODIUM
00012750	Matulane	PROCARBAZINE HCL

02403390	Stivarga	REGORAFENIB
02473569	Kisqali	RIBOCICLIB SUCCINATE
02388006	Jakavi	RUXOLITINIB
02388014	Jakavi	RUXOLITINIB
02388022	Jakavi	RUXOLITINIB
02434814	Jakavi	RUXOLITINIB
02284227	Nexavar	SORAFENIB
02280795	Sutent	SUNITINIB MALATE
02280809	Sutent	SUNITINIB MALATE
02280817	Sutent	SUNITINIB MALATE
00812390	Apo-Tamox	TAMOXIFEN CITRATE
00812404	Apo-Tamox	TAMOXIFEN CITRATE
00839353	ALPHA-TAMOXIFEN	TAMOXIFEN CITRATE
00839361	ALPHA-TAMOXIFEN	TAMOXIFEN CITRATE
00851965	Teva-Tamoxifen	TAMOXIFEN CITRATE
00851973	Teva-Tamoxifen	TAMOXIFEN CITRATE
01926624	Tamofen	TAMOXIFEN CITRATE
01926632	Tamofen	TAMOXIFEN CITRATE
02048485	Nolvadex D	TAMOXIFEN CITRATE
02088428	Mylan-Tamoxifen	TAMOXIFEN CITRATE
02089858	Mylan-Tamoxifen	TAMOXIFEN CITRATE
02241093	Temodal	TEMOZOLOMIDE
02241094	Temodal	TEMOZOLOMIDE
02241095	Temodal	TEMOZOLOMIDE
02241096	Temodal	TEMOZOLOMIDE
02312794	Temodal	TEMOZOLOMIDE
02312816	Temodal	TEMOZOLOMIDE
02395274	Co Temozolomide	TEMOZOLOMIDE
02395282	Co Temozolomide	TEMOZOLOMIDE
02395290	Co Temozolomide	TEMOZOLOMIDE
02395312	Co Temozolomide	TEMOZOLOMIDE
02441160	Act Temozolomide	TEMOZOLOMIDE
02443473	Taro-Temozolomide	TEMOZOLOMIDE
02443481	Taro-Temozolomide	TEMOZOLOMIDE
02443511	Taro-Temozolomide	TEMOZOLOMIDE
02443538	Taro-Temozolomide	TEMOZOLOMIDE
02443554	Taro-Temozolomide	TEMOZOLOMIDE
02472104	Lonsurf	TIPIRACIL HCL & TRIFLURIDINE
02472112	Lonsurf	TIPIRACIL HCL & TRIFLURIDINE
02355191	Thalomid	THALIDOMIDE
02355205	Thalomid	THALIDOMIDE

02355221	Thalomid	THALIDOMIDE
00282081	Lanvis	THIOGUANINE
02246016	Thyrogen	THYROTROPIN ALFA
02409623	Mekinist	TRAMETINIB
02409658	Mekinist	TRAMETINIB
02240000	Trelstar (1 Month)	TRIPTORELIN PAMOATE
02243856	Trelstar LA (3 Month)	TRIPTORELIN PAMOATE
02412322	Trelstar	TRIPTORELIN PAMOATE
09857199	Trelstar (1 Month)	TRIPTORELIN PAMOATE
09857200	Trelstar LA (3 Month)	TRIPTORELIN PAMOATE
02378582	Caprelsa	VANDETANIB
02378590	Caprelsa	VANDETANIB
02380242	Zelboraf	VEMURAFENIB
02458039	Venclexta	VENETOCLAX
02458047	Venclexta	VENETOCLAX
02458055	Venclexta	VENETOCLAX
02458063	Venclexta	VENETOCLAX
00611182	Oncovin	VINCRISTINE SULFATE
02143305	Vincristine Sulfate	VINCRISTINE SULFATE
02409267	Erivedge	VISMODEGIB
02327619	Zolinza	VORINOSTAT

Supplementary Table S2. Active Cancer Diagnosis Definition Codes

Ontario Health Insurance Plan to define chemotherapy	G281, G339, G345, G359, G381, G382, G075, G390, G388
Ontario Health Insurance Plan to define radiation	X310, X311, X312, X313, X322, X323, X324, X325, X334, X305, X306
Canadian Classification of Health Interventions codes to define cancer surgery	1NK77, 1NM77, 1AN87, 1AJ87, 1AC87, 1AP87, 1AA87, 1AW87, 1EA87, 1YM87, 1MD87, 1NM87, 1NQ87, 1NK87, 1OA87, 1OT87, 1MH87, 1MG87, 1RM87, 1MC87, 1FJ87, 1FH87, 1EE87, 1ED87, 1FM87, 1FG87, 1FX87, 1GR87, 1GT87, 1YF87, 1YS87, 1YV87, 1YA87, 1YG87, 1YW87, 1VX87, 1EQ87, 1TX87, 1SZ87, 1SH87, 1WV87, 1DA87, 1YB87, 1YD87, 1NF87, 1OK87, 1OA87, 1NK87, 1OJ87, 1NA87, 1PM87, 1PC87, 1PG87, 1PL87, 1QE87, 1FU87, 1YM88, 1NA88, 1YM91, 1NM91, 1MC91, 1FJ91, 1GE91, 1EE91, 1FM91, 1ED91, 1EN91, 1GT91, 1GR91, 1MC91, 1MJ91, 1PC91, 1PM91, 1QM91, 1QT91, 1NF91, 1FX91, 1YM92, 1EA92, 1PM92, 1NF92, 1YM89, 1MD89, 1NQ89, 1NM89, 1RD89, 1RM89, 1MC89, 1FM89, 1GR89, , 1GT89, 1GV89, 1MD89, 1MJ89, 1MC89, 1MH89, 1OD89, 1OE89, 1NF89, 1NA89, 1MH89, 1MG89, 1PQ89, 1QM89, 1PG89, 1FU89, 1OB89, 1GE89, 1YM90, 1NA90, 1NF90
